# Emerging Therapeutic Strategies for Attenuating Tubular EMT and Kidney Fibrosis by Targeting Wnt/β-Catenin Signaling

**DOI:** 10.3389/fphar.2021.830340

**Published:** 2022-01-10

**Authors:** Lichao Hu, Mengyuan Ding, Weichun He

**Affiliations:** Center for Kidney Disease, Second Affiliated Hospital, Nanjing Medical University, Nanjing, China

**Keywords:** epithelial-mesenchymal transition, tubular epithelial cell, Wnt/β-catenin signaling, kidney fibrosis, myofibroblast

## Abstract

Epithelial-mesenchymal transition (EMT) is defined as a process in which differentiated epithelial cells undergo phenotypic transformation into myofibroblasts capable of producing extracellular matrix, and is generally regarded as an integral part of fibrogenesis after tissue injury. Although there is evidence that the complete EMT of tubular epithelial cells (TECs) is not a major contributor to interstitial myofibroblasts in kidney fibrosis, the partial EMT, a status that damaged TECs remain inside tubules, and co-express both epithelial and mesenchymal markers, has been demonstrated to be a crucial stage for intensifying fibrogenesis in the interstitium. The process of tubular EMT is governed by multiple intracellular pathways, among which Wnt/β-catenin signaling is considered to be essential mainly because it controls the transcriptome associated with EMT, making it a potential therapeutic target against kidney fibrosis. A growing body of data suggest that reducing the hyperactivity of Wnt/β-catenin by natural compounds, specific inhibitors, or manipulation of genes expression attenuates tubular EMT, and interstitial fibrogenesis in the TECs cultured under profibrotic environments and in animal models of kidney fibrosis. These emerging therapeutic strategies in basic researches may provide beneficial ideas for clinical prevention and treatment of chronic kidney disease.

## Introduction

Kidney fibrosis, a pathological process characterized by excessive deposition of extracellular matrix (ECM) in the interstitium accompanied by destruction of normal kidney architecture, is a hallmark and inevitable end point of all kinds of progressive chronic kidney disease (CKD). Myofibroblast is well known as the major type of matrix-producing cell, the source of which has long been controversial and remains a hot area of research in nephrology (Mack and Yanagita, 2015; Yuan et al., 2019). Based on much of the current data from studies using lineage tracing techniques, the main origins of myofibroblasts are accepted to be resident mesenchymal cells including fibroblast (Asada et al., 2011; Lebleu et al., 2013), pericyte (Humphreys et al., 2010; Gomez and Duffield, 2014), and mesenchymal stem cell (Kramann et al., 2015; El Agha et al., 2017; Kramann et al., 2017), and other precursors comprising circulating bone marrow-derived progenitor (Li et al., 2007; Lebleu et al., 2013), peritubular endothelial cell (Zeisberg et al., 2008; Cruz-Solbes and Youker, 2017), and tubular epithelial cell (TEC) (Lebleu et al., 2013; Cruz-Solbes and Youker, 2017).

TEC, as a major component of renal parenchyma, is particularly vulnerable to damage during acute kidney injury (AKI) and is also a driving force for the progression of CKD. The involvement of epithelial-mesenchymal transition (EMT) in kidney fibrosis is widely concerned (Liu et al., 2018). EMT is traditionally defined as a phenotypic conversion programme in which the damaged TEC loses epithelial markers and acquires mesenchymal features (Liu, 2010; Cruz-Solbes and Youker, 2017). The contribution of this complete EMT to interstitial myofibroblasts appears to be very low (Lebleu et al., 2013), whereas the partial EMT is of more concern (Zhou and Liu, 2016; Sheng and Zhuang, 2020). Partial EMT refers to a status in which damaged TECs express both markers of epithelial and mesenchymal but remain inside tubules with G2/M phase cell cycle arrest, resulting in compromised regeneration and repair, impaired functionality, and altered secretome. EMT begins with stress responses of TEC to protect from damage and ultimately allows cells to acquire a secretory phenotype, leading to the release of pathological mediators that persistently activate various myofibroblast precursors. Partial EMT has been demonstrated to be an indispensable stage of fibrogenic progression, making inhibition of EMT one of the main strategies for restraining kidney fibrosis (Liu, 2010; Grande et al., 2015; Lovisa et al., 2015; Zhou and Liu, 2016).

EMT process is governed by sophisticated signal networks involving several developmental pathways, such as Wnt, Notch, and Hedgehog. Of them, the role of Wnt/β-catenin signaling is believed to be essential. Numerous studies have demonstrated that Wnt/β-catenin signaling is a potent mediator of EMT process. Hence, the intervention in EMT via modulating activity of this pathway is considered a promising therapeutic strategy against kidney fibrosis. In this mini review, we briefly discuss the mechanisms by which Wnt/β-catenin signaling regulates tubular EMT process, and summarize current strategies to interfere with EMT by modulating activity of this signaling.

## Wnt/β-Catenin and Tubular EMT

In canonical Wnt cascade, when Wnt ligands bind to receptors Frizzled protein (FZD) and lipoprotein receptor-related protein-5 or 6 (LRP5/6), Disheveled protein (Dvl) is recruited and a cytoplasmic destruction complex comprising proteins adenomatous polyposis coli (APC), Axin, casein kinase 1 (CK1), and glycogen synthase kinase 3β (GSK3β) is inhibited, resulting in de-phosphorylation, stabilization, and nuclear translocation of β-catenin. In the nucleus, the combination of β-catenin with T-cell factor and lymphoid enhancer-binding factor (TCF/LEF) initiates transcription of Wnt target genes (Clevers and Nusse, 2012; Nusse and Clevers, 2017). Wnt/β-catenin signaling seems quiescent in normal adult kidneys, whereas in injured kidneys, Wnt proteins are markedly induced. Transient activation of Wnt/β-catenin signaling favors cell regeneration and tissue repair after AKI, but its sustained activation aggravates kidney fibrosis in CKD progression (Zhou et al., 2013a; Zhou et al., 2013b; Tan et al., 2014; Schunk et al., 2021).

TECs are a main source of Wnt proteins in injured kidneys, and these ligands act in an autocrine or paracrine manner between several cell types. Activation of Wnt/β-catenin signaling induces transformation of TECs into a secretory phenotype with most partial EMT and a few complete EMT, induces proliferation, activation and differentiation of interstitial fibroblasts into myofibroblasts, and induces polarization M2 phenotype, and pro-inflammatory activation of macrophages. In turn, Wnt ligands derived from fibroblasts and macrophages can also target TECs directly (Schunk et al., 2021).

In TECs, Wnt/β-catenin signaling takes effects by inducing its target genes, some of which play the substantial roles in regulating tubular EMT process in the setting of CKD, such as fibroblast-specific protein 1 (FSP-1), fibronectin, matrix metalloproteinase 7 (MMP7), Snail, and Twist (Boutet et al., 2006; He et al., 2009; He et al., 2012; Tan et al., 2014; Ning et al., 2018). Concretely, FSP-1 and fibronectin are commonly used as EMT markers because they are not normally expressed in epithelial cells. FSP-1 is a marker for myofibroblasts, while fibronectin is a major component of ECM (Liu, 2011). MMP7, a secreted zinc- and calcium-dependent endopeptidase that acts on a variety of substrates to regulate various cellular processes, is a critical regulatory factor in EMT by mediating E-cadherin ectodomain shedding and proteolytic degradation (He et al., 2012; Zhou et al., 2017a; Liu et al., 2020). Snail and Twist are critical transcription factors that drive EMT programme. Conditional deletion of Snail or Twist1 in TECs inhibited EMT programme, which in turn alleviated interstitial fibrosis in several CKD models (Grande et al., 2015; Lovisa et al., 2015). We will briefly discuss the functions of the two proteins in controlling EMT in detail.

Snail is a member of the zinc finger 1 transcription factor family and is able to trigger the first step in EMT process by transcriptionally suppressing the expression of E-cadherin and disrupting adhesions between epithelial cells (Cano et al., 2000; Liu, 2004; Hao et al., 2011; Simon-Tillaux and Hertig, 2017). By repressing E-cadherin, Snail also releases β-catenin from the dissociating adherens junctions, thus further facilitating the cell to EMT programme, because in addition to intracellular β-catenin that can act as a signaling sensor after Wnt signal activation, β-catenin located near the cytoplasmic membrane can physically interact with E-cadherin (Wang et al., 2010). Moreover, Snail activates the production of alpha smooth muscle actin (α-SMA) and vimentin, two mesenchymal markers (Cano et al., 2000; Boutet et al., 2006), and induces Id1, a transcription antagonist that plays a crucial role in promoting EMT (Li et al., 2012). Besides being a transcriptional target of Wnt/β-catenin, Snail is post-transcriptionally modified by GSK3β and can cooperate with Wnt ligands to induce the signaling. Thus, when Wnt ligands initiate the signaling, the simultaneous activation of β-catenin, and Snail produces synergistic or additive effects in driving EMT (García de Herreros and Baulida, 2012; Schunk et al., 2021). In addition to EMT programming, Snail also controls other major biological processes responsible for renal fibrogenesis, such as interference of fatty acid metabolism, cell cycle arrest, and inflammation (Simon-Tillaux and Hertig, 2017). Furthermore, Snail-induced partial EMT could orchestrate p53-p21-mediated G2/M arrest via nuclear factor kappa B-mediated inflammation in CKD models (Qi et al., 2021).

Twist is a transcription factor of the basic helix-loop-helix class and is capable of not only repressing E-cadherin gene transcription by binding to the E-boxes in its promoter region but also inducing the expression of mesenchymal markers including fibronectin, vimentin, α-SMA, and N-cadherin (Howe et al., 2003; Yang et al., 2004; Kida et al., 2007). Additionally, Twist also regulates hypoxia-induced EMT in a hypoxia inducible factor-1 (HIF-1)-dependent manner in renal fibrosis (Bechtel and Zeisberg, 2009; Sun et al., 2009). Bmi1 is responsible for Twist1-induced EMT (Yang et al., 2010), and the promoter of Bmi1 contains potential binding sites for Twist1 and HIF-1α. Under hypoxic conditions, Twist1 and HIF-1α cooperatively enhanced Bmi1 transcriptional activation and controlled its downstream target genes including Snail and E-cadherin (Du et al., 2014; Ning et al., 2018).

In a short, sustained activation of Wnt/β-catenin signaling is a potent propeller of EMT. Therefore, it represents a promising therapeutic target to restrain tubular EMT process and mitigate kidney fibrosis.

## Emerging Strategies to Suppress EMT by Targeting Wnt/β-Catenin

A great deal of strategies for hampering tubular EMT process and alleviating kidney fibrosis through inhibiting the activity of Wnt/β-catenin signaling in various animal or cellular models of CKD have been reported, in which the components of Wnt/β-catenin pathway were selectively or specifically detected for exploring the intrinsic relationship between the strategy and the change in the activity of the signaling. These studies are shown below based on the most upstream level of the components of the signaling pathway being examined and are summarized in Table 1.

**TABLE 1:** Potential modulations for inhibiting EMT and components of Wnt/β-catenin pathway involved.

Modulators or modulation methods for inhibiting tubular EMT	Factors that induce tubular EMT	Experimental models of CKD	Components of the pathway involved that are detected	References
Downregulation of β-Arrestin-1	β-arrestin-1; TGFβ1	UUO mice; TGFβ1-treated HK-2 cells	Wnt1, active β-catenin	Xu et al. (2018)
AGER1; Downregulation of RAGE; ICG-001	AOPP; Downregulation of AGER1	AOPP-treated HKC-8 cells	Wnt1, p-GSK3β, β-catenin, TCF4	Feng et al. (2020)
Overexpression of SIK1; Downregulation of β-catenin; Downregulation of Twist1	AA	AA-induced AKI-CKD transition mice; AA-treated HK-2 cells	Wnt1, p-β-catenin (Y654), nuclear β-catenin, Snail, Twist1	Hu et al. (2021)
U0126 (ERK1/2 inhibitor); Downregulation of ERK1/2	Uric acid	Hyperuricemic nephropathy rats	Wnt1, β-catenin	Liu et al. (2017), Tao et al. (2019)
Downregulation of MMP2; Minocycline (MMP inhibitor)	MMP2	UUO mice	Wnt1, β-catenin, Snail	Du et al. (2012)
25-O-methylalisol F (MAF)	TGFβ1; ANG	TGFβ1- or ANG-treated NRK-52E cells	Wnt1, active β-catenin, Snail1, Twist, MMP7, PAI-1, FSP-1	Chen et al. (2018a)
Vitexin	COM; Glyoxylate	Glyoxylate-induced nephrolithiasis mice; COM-treated HK-2 cells	Wnt1, p-β-catenin, β-catenin	Ding et al. (2021)
Astragaloside IV (AS-IV)	HG	Type 2 DKD rats; HG-treated HK-2 cells	Wnt1, β-catenin, nuclear β-catenin, GSK3β-APC-Axin protein complex	Wang et al. (2020)
Atractylenolide I (ATL-1)	TGFβ1	UUO mice; TGFβ1-treated NRK-52E cells	Wnt1, p-β-catenin/β-catenin	Guo et al. (2021)
Downregulation of WISP1	Uremia	Uremic rats	Wnt2b, c-Myc, cyclin D	Chen et al. (2019)
Downregulation of CRP	CRP; TGFβ1	STZ-induced DKD rats; TGFβ1 or CRP-treated HK-2 cells	Wnt3a, β-catenin	Zhang et al. (2019)
Overexpression of kallistatin	Downregulation of kallistatin; TGFβ1	TGFβ1-treated HK-2 cells; UUO mice	Wnt4, DKK1, Axin2, p-GSK3β (Ser9)/GSK3β, β-catenin, active β-catenin, fibronectin, Snail, PAI-1, Renin	Yiu et al. (2021)
Anti-FKN antibody; XAV939 (β-catenin inhibitor)	FKN; ANG	MRL/lpr mice; ANG-treated HK-2 cells	Wnt4, β-catenin, c-Myc, cyclin D1	Fu et al. (2019)
Downregulation of RSPO1; Downregulation of LGR4	RSPO1	High fat diet-induced obesity mice; Recombinant RSPO1-treated HK-2 cells	LRP6, p-GSK3β (Ser9)/GSK3β, active β-catenin, nuclear β-catenin	Carmon et al., 2011, Su et al., 2021
Overexpression of CFTR; iCRT14 (β-catenin inhibitor)	CFTR inhibitor (CFTRinh-172 or GlyH101); downregulation of CFTR	UUO mice; Hypoxia-treated MDCK cells and HK-2 cells	Dvl2, nuclear β-catenin, Axin2, Met, MMP7, MMP2, cyclin D2	Zhang et al. (2017)
Downregulation of DOCK4; Downregulation of USP36	USP36; HG	STZ-induced DKD mice; HG-treated HK-2 cells	β-catenin degradation complex, β-catenin	Zhu et al. (2021)
Overexpression of AMPKα2	Downregulation of AMPKα2	UUO mice; HKC cells with downregulated AMPKα2	β-catenin	Qiu et al. (2015)
Downregulation of FHL2	Overexpression of FHL2; TGFβ1	UUO mice; TGFβ1-treated NRK-52E cells	Active β-catenin, nuclear β-catenin, Snail, Twist, vimentin, PAI-1, MMP7	Cai et al. (2018)

RAGE, receptor of advanced glycation end-products; AGER1, advanced glycation end-products receptor 1; AOPP, advanced oxidative protein product; SIK1, salt inducible kinase 1; AA, aristolochic acid; ERK, extracellular signal-regulated kinase; MMP, matrix metalloproteinase; ANG, angiotensin II; COM, calcium oxalate monohydrate; HG, high glucose; DKD, diabetic kidney disease; GSK3β, glycogen synthase kinase-3β; APC, adenomatous polyposis coli; WISP1, Wnt-inducible signaling pathway protein-1; CRP, C-reactive protein; STZ, streptozotocin; FKN, fractalkine; RSPO1, R-spondin 1; LGR4, leucine-rich repeat-containing G protein coupled receptor 4; CFTR, cystic fibrosis transmembrane conductance regulator; MDCK, renal distal tubular Madin-Darby canine kidney; DOCK4, dedicator of cytokinesis 4; USP36, ubiquitin specific proteases 36; AMPK, AMP-activated protein kinase; FHL2, four and a half LIM domain protein 2.

### Wnt1

β-Arrestin-1 is a negative adapter of G-protein-coupled receptors (GPCRs) and also acts as a scaffold protein that regulates various cellular functions independently of GPCR activation (Kendall and Luttrell, 2009). Xu et al. reported that β-arrestin-1 was induced in the fibrotic kidneys in mice with unilateral ureteral obstruction (UUO) and in the TGFβ1-treated TECs and renal fibroblasts. Gene silencing of β-arrestin-1 reduced EMT and fibroblasts activation and attenuated kidney fibrosis, as well as diminished the upregulation of Wnt1 mRNA and active β-catenin *in vivo* and *in vitro* (Xu et al., 2018).

Advanced oxidative protein product (AOPP), belonging to dityrosine-containing protein family, is a marker of protein glycoxidation closely related to oxidative stress. As a uremic toxin, AOPP has been found accumulation in patients with CKD. Chronic accumulation of AOPP aggravated kidney fibrosis in animal models (Shi et al., 2008). Feng et al. reported that AOPP induced EMT through activating receptor of advanced glycation end-products (RAGE)/Wnt/β-catenin pathway in the cultured TECs. Either ICG-001, an inhibitor of β-catenin, or RAGE knockout, or advanced glycation end-products receptor 1 (AGER1, an antagonist of RAGE), could inhibit AOPP-induced EMT. AOPP-induced upregulation of Wnt1, p-GSK3β, β-catenin, and TCF4 was suppressed by downregulation of RAGE (Feng et al., 2020).

Salt inducible kinase 1 (SIK1), a member of AMP-activated protein kinases (AMPKs) family, plays a key role in regulating metabolism, cell survival, and growth (Taub, 2019). Hu et al. reported that the expression of SIK1 was downregulated in the kidneys from mice with AKI-CKD transition induced by aristolochic acid (AA) and in AA-treated TECs, whereas upregulation of SIK1 alleviated EMT, inflammation and fibrogenesis, and impeded AKI-CKD transition. Mechanistically, overexpression of SIK1 inhibited AA-induced upregulation of Wnt1 and p-β-catenin (Y654), the increase in β-catenin nuclear translocation, and the upregulation of Snail and Twist1 (Hu et al., 2021).

Extracellular signal-regulated kinase-1 and 2 (ERK1/2) are serine/threonine kinases that have been found to be involved in uric acid-mediated EMT and the pathogenesis of hyperuricemic nephropathy (HN) (Liu et al., 2017). Tao et al. reported that inhibition of ERK1/2 by either U0126, a selective inhibitor of ERK1/2 pathway, or specific siRNA, mitigated EMT in the kidneys from HN rats through inactivation of multiple signaling pathways including Wnt/β-catenin. The induction of Wnt1 and β-catenin was remarkably suppressed by inhibition of ERK1/2 (Tao et al., 2019).

MMPs are a family of zinc-dependent proteases, and MMPs-mediated destruction of tubule basement membrane integrity was once believed to be a key step in promoting EMT (Cheng and Lovett, 2003; Cheng et al., 2006). Although the complete EMT is no longer considered a major contributor to interstitial myofibroblasts, MMPs still play a role in fibrogenesis. Du et al. reported that the activities of MMP2 and MMP9 were increased in the kidneys from UUO mice, while inactivation of MMP2 by either MMP2 knockout or minocycline, an inhibitor of MMPs, suppressed inflammation and EMT, and ameliorated kidney fibrosis. The upregulation of Wnt1, β-catenin, and Snail in the UUO kidneys were restrained by inhibition of MMP2 (Du et al., 2012).

Triterpenoid compounds are main active components in *Alismatis rhizoma*, a natural product with lipid-lowering and renoprotective effects (Tian et al., 2014; Ma et al., 2016). Chen et al. reported that 25-O-methylalisol F (MAF), a new triterpenoid compound, was able to inhibit TGFβ1- or angiotensin II (ANG)-induced EMT in TECs and renal fibroblast activation, respectively. The effect of MAF on EMT was related to its regulation of renin-angiotensin system, TGFβ1/Smad, and Wnt/β-catenin. TGFβ1- or ANG-induced upregulation of Wnt1, active β-catenin and downstream targets Snail1, Twist, MMP7, PAI-1, and FSP-1 were inhibited by MAF (Chen et al., 2018a).

Recurrent nephrolithiasis is a contributor to kidney fibrosis, and the pathogenesis involves oxidative stress, inflammation, apoptosis, and EMT (Khan et al., 2016). Vitexin (apigenin-8-C-β-D-glucopyranoside), a flavonoid monomer derived from *Ficus deltoidea*, bamboo, and dried hawthorn leaves, possesses biological effects including antivirus, anti-inflammatory, and anticancer (Xue et al., 2020; Yahaya et al., 2020). Ding et al. reported that vitexin alleviated crystal deposition and kidney injury in a mouse model of nephrolithiasis induced by glyoxylate and cell models of TECs and macrophages treated with calcium oxalate monohydrate (COM), and the protective role of vitexin was related to the inhibition of pyroptosis, apoptosis, EMT, and macrophage infiltration. The upregulation of Wnt1 and β-catenin and downregulation of p-β-catenin in COM-treated TECs were restrained by vitexin (Ding et al., 2021).

Astragaloside IV (AS-IV), a saponin extracted from *Astragalus membranaceus*, possesses rich pharmacological activities, including antioxidant stress, anti-inflammatory, anti-diabetes, and renal protection (Fu et al., 2014; Zhou et al., 2017b; Chen et al., 2018b). Wang et al. reported that AS-IV repressed EMT, fibrogenesis, oxidative stress, and inflammation by inactivating Wnt/β-catenin signaling in a rat model of type 2 diabetic kidney disease (DKD) and in high glucose (HG)-treated TECs. HG-induced upregulation of Wnt1 and β-catenin and an increase in nuclear β-catenin were inhibited by AS-IV. In addition, AS-IV could regulate the activity of Wnt/β-catenin signaling *via* binding to GSK3β-APC-Axin protein complex (Wang et al., 2020).

Atractylenolide I (ATL-1), a eudesmane-type sesquiterpenoid lactone derivative of *Rhizoma Atractylodis macrocephalae*, possesses various biological activities including antioxidant and anticancer (Li et al., 2020). Guo et al. reported that ATL-1 inhibited EMT and fibroblasts activation in the kidneys from UUO mice and in TGFβ1-treated TECs and renal fibroblasts. ATL-1 suppressed the activities of several proliferation-related pathways including Wnt/β-catenin. The upregulation of Wnt1 and the decrease in p-β-catenin/β-catenin ratio in UUO kidneys were restrained by ATL-1 (Guo et al., 2021).

### Wnt2b

Wnt-inducible signaling pathway protein-1 (WISP1, also known as CCN4), belonging to the CCN family of ECM proteins, is a downstream target of Wnt/β-catenin, and has been shown to be involved in fibrotic diseases (Murahovschi et al., 2015). Chen et al. reported that the expression of WISP1 was induced and Wnt/β-catenin signaling was activated in the kidneys from a rat model of uremia, while WISP1 gene silencing repressed tubular EMT through inhibiting Wnt/β-catenin signaling. The upregulation of Wnt2b, c-Myc, and cyclin D1 in uremia is inhibited by WISP1 deficiency (Chen et al., 2019).

### Wnt3a

C-reactive protein (CRP), an acute phase plasma protein, is generally considered as a non-specific marker of inflammation (Pepys and Hirschfield, 2003), however, many studies have confirmed that CRP is involved in the pathogenesis of many diseases (Szalai, 2004; Zhang et al., 2015). Elevated CRP expression level has been found in DKD and CKD (Menon et al., 2005; Hayashino et al., 2014). Zhang et al. reported that CRP enhanced EMT in the kidneys from STZ-induced DKD rats and in the TGFβ1-treated TECs, and the effects of CRP on EMT involved Wnt/β-catenin and ERK signaling. CRP facilitated the upregulation of Wnt3a and β-catenin induced by TGFβ1 in TECs, whereas deficiency of CRP inhibited the induction of Wnt3a and β-catenin *in vivo* (Zhang et al., 2019).

### Wnt4

Kallistatin is a serine protease inhibitor that regulates multiple pathways involving in various biological functions such as vasodilation, angiogenesis, oxidative stress, inflammation, and fibrosis (Huang et al., 2014; Chao et al., 2016; Yiu et al., 2016; Wang et al., 2017; Guo et al., 2018). Yiu et al. reported that kallistatin levels were markedly lower in the kidneys from CKD patients. In UUO mice, depletion of endogenous kallistatin resulted in aggravated tubular EMT and kidney fibrosis, while overexpression of kallistatin exerted kidney protective effects. Depletion of kallistatin increased the levels of Wnt4, p-GSK3β (Ser9)/GSK3β, Axin2, active β-catenin, and target genes of Wnt/β-catenin, whereas overexpression of kallistatin restrained the activation of Wnt/β-catenin. The regulatory effect of kallistatin on EMT and the activity of Wnt/β-catenin pathway in TGFβ1-treated TECs was similar to that *in vivo* (Yiu et al., 2021).

Fractalkine (FKN), also known as chemokine (C-X3-C motif) ligand 1, is a chemokine that regulates cell adhesion and growth and has been shown to be involved in the pathogenesis of inflammatory diseases including autoimmune disease (Ruchaya et al., 2014; Liao et al., 2017). Fu et al. reported that FKN was induced in the kidneys from MRL/lpr mice (a murine model of lupus nephritis). Treatment with an anti-FKN antibody suppressed EMT and fibrogenesis and improved renal function along with suppressing the activation of Wnt/β-catenin signaling, whereas the administration of recombinant FKN exhibited the opposite effects. The effect of FKN on EMT and the activation of Wnt/β-catenin in ANG-treated TECs was similar to that *in vivo*. Inactivation of Wnt/β-catenin by an antagonist XAV939 blockaded the enhancement of FKN overexpression to the EMT. The affected components of the pathway by FKN included Wnt4, β-catenin, c-Myc, and cyclin D1 (Fu et al., 2019).

### LRP6

R-spondin1 (RSPO1), a member of secretory protein RSPOs family, possesses a high affinity with leucine-rich repeat-containing G protein coupled receptor 4 (LGR4). RSPO1 has been identified as an activator of Wnt/β-catenin signaling because the binding of LGR4 and RSPO1 enhanced Wnt-induced phosphorylation of LRP6 (Carmon et al., 2011). It has been found that circulating RSPO1 was remarkably elevated in patients with obesity and insulin resistant (Kang et al., 2019). Su et al. reported that the expression of RSPO1 was induced in the kidneys from obesity mice fed with high-fat diet, while knockdown of RSPO1 alleviated kidney injury and fibrogenesis. The recombinant RSPO1 facilitated EMT process by binding to LGR4 to activate Wnt/β-catenin signaling, represented by an increase in active β-catenin and nuclear β-catenin in TECs, whereas these effects of RSPO1 could be diminished by downregulation of LGR4 (Su et al., 2021).

### Degradation Complex

Cystic fibrosis transmembrane conductance regulator (CFTR), a cAMP-activated Cl^−^ channel, is abundantly expressed at the apical surfaces of proximal, and distal tubules in normal kidneys (Kibble et al., 2000; Morales et al., 2000). Zhang et al. reported that the expression of CFTR was downregulated in the fibrotic kidneys from both CKD patients and UUO mice and in the TECs cultured under hypoxia condition. Suppression of CFTR function or expression by CFTR inhibitor, CFTRinh-172 or GlyH101, is sufficient to trigger EMT process *in vitro*. Knockdown of CFTR increased nuclear β-catenin, enhanced β-catenin-mediated transcriptional activity, and upregulated the expression of target genes, whereas iCRT14, a β-catenin inhibitor, blocked the effect of CFTR downregulation on EMT. Mechanistically, the interaction of CFTR and Dvl2 *via* PDZ domain appears to contribute to the inhibitory effect of CFTR on β-catenin activity (Zhang et al., 2017).

Dedicator of cytokinesis 4 (DOCK4), a guanine nucleotide exchange factor for Rac, has been reported to enhance the stability and activity of β-catenin and induce EMT by interacting with β-catenin degradation complex to increase the level of cellular β-catenin response to Wnt ligands (Upadhyay et al., 2008; Xie et al., 2020). Zhu et al. reported that the expression of ubiquitin specific proteases 36 (USP36), a member of deubiquitinating enzymes family, was induced in DKD in human and murine model and in HG-treated TECs, and the overexpression of USP36 enhanced EMT in TECs. Additionally, USP36 directly bound to and mediated the de-ubiquitination of DOCK4, whereas DOCK4 knockdown effectively abolished EMT induced by USP36 overexpression through suppressing Wnt/β-catenin signaling in TECs (Zhu et al., 2021).

### β-Catenin

AMPK, a heterotrimeric serine/threonine protein kinase, functions as an energy sensor in response to stresses, and regulates cell energy balance and differentiation (Mihaylova and Shaw, 2011; Ruderman et al., 2013). Qiu et al. reported that knockdown of AMPKα, especially AMPKα2, enhanced EMT by activating Wnt/β-catenin and TGFβ/Smad signaling in TECs, and AMPKα2 deficiency exacerbated EMT and inflammation and promoted fibrogenesis in the kidneys from UUO mice. The results in this study demonstrated that AMPKα2 was able to decrease the expression of β-catenin in TECs (Qiu et al., 2015).

Four and a half LIM domain protein 2 (FHL2) belongs to the members of FHL subfamily that is included in LIM-only proteins family. FHL2 acts as a scaffold protein interacting with various intracellular protein partners, enabling it to regulate signaling pathways that involve a plethora of cellular tasks (Tran et al., 2016). We have reported that FHL2 was upregulated in the fibrotic kidneys in CKD patients and in UUO mice and in the TGFβ1-treated TECs (Cai et al., 2018; Duan et al., 2020). Overexpression of FHL2 promoted EMT, whereas downregulation of FHL2 suppressed EMT induced by TGFβ1. The interaction between FHL2 and β-catenin in TECs was increased by TGFβ1, and knockdown of FHL2 increased β-catenin phosphorylation and decreased nuclear localization of β-catenin, β-catenin-mediated transcription and its target genes expression (Cai et al., 2018).

## Discussion

Although TECs undergoing conventional EMT are no longer recognized as a major constituent of interstitial myofibroblasts, the partial EMT has been demonstrated to exert the crucial functions in the fibrogenesis during kidney fibrosis progression. Given the importance of Wnt/β-catenin signaling in the regulation of EMT, targeting this pathway to restrain tubular EMT process has become a promising strategy for inhibiting kidney fibrosis, which has attracted numerous researchers to conduct relevant studies.

**FIGURE 1:**
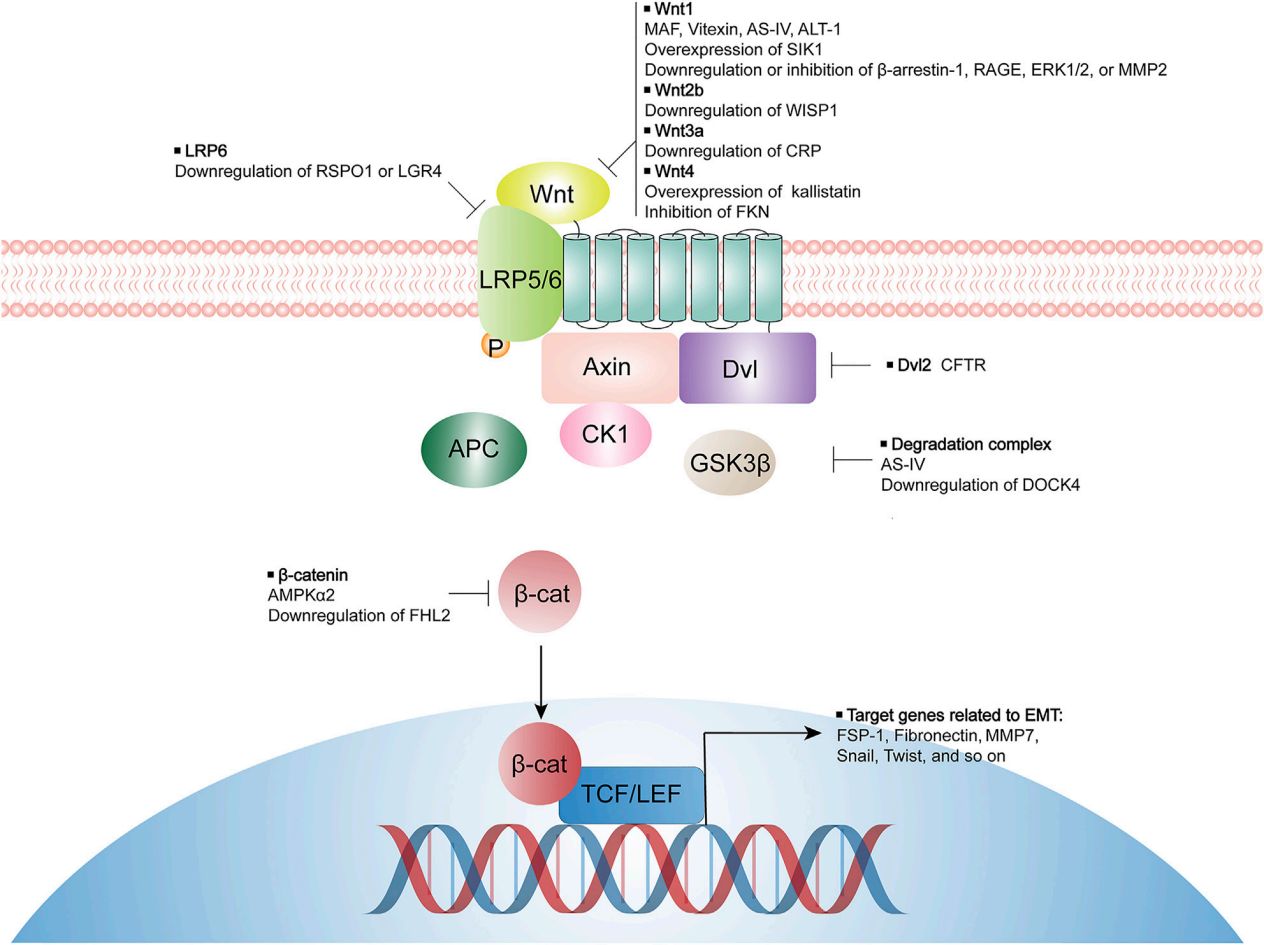
Modulators for inhibiting tubular EMT and their potential targeted components of Wnt/β-catenin pathway. LRP, lipoprotein receptor-related protein; Dvl, disheveled; GSK3β, glycogen synthase kinase-3β; APC, adenomatous polyposis coli; MAF, 25-O-methylalisol F; AS-IV, Astragaloside IV; ATL-1, Atractylenolide I; SIK1, salt inducible kinase 1; RAGE, receptor of advanced glycation end-products; ERK, extracellular signal-regulated kinase; MMP, matrix metalloproteinase; WISP1, Wnt-inducible signaling pathway protein-1; CRP, C-reactive protein; FKN, fractalkine; RSPO1, R-spondin 1; LGR4, leucine-rich repeat-containing G protein coupled receptor 4; CFTR, cystic fibrosis transmembrane conductance regulator; DOCK4, dedicator of cytokinesis 4; AMPK, AMP-activated protein kinase; FHL2, four and a half LIM domain protein 2.

Data from animal models and cell experiments suggest that inhibiting Wnt/β-catenin signaling activity by either natural compounds, specific inhibitors, or manipulation of selective genes expression, may effectively suppress tubular EMT process and mitigate kidney interstitial fibrosis. Some of these studies have deeply investigated the mechanism by which Wnt/β-catenin activity is inhibited, while others have only observed the inhibitory effect of certain modulators on Wnt/β-catenin activity but not the mechanism of the signaling activity inhibition. Potential targeted components of Wnt/β-catenin pathway by various modulators are summarized in Figure 1. In conclusion, targeting Wnt/β-catenin signaling precisely to impede EMT process remains a challenge but one that carries great opportunities for the inhibition of kidney fibrosis and the therapy of CKD.
